# QseC Mediates Osmotic Stress Resistance and Biofilm Formation in *Haemophilus parasuis*

**DOI:** 10.3389/fmicb.2018.00212

**Published:** 2018-02-13

**Authors:** Lvqin He, Ke Dai, Xintian Wen, Lingqiang Ding, Sanjie Cao, Xiaobo Huang, Rui Wu, Qin Zhao, Yong Huang, Qigui Yan, Xiaoping Ma, Xinfeng Han, Yiping Wen

**Affiliations:** ^1^Research Center of Swine Disease, College of Veterinary Medicine, Sichuan Agricultural University, Chengdu, China; ^2^Sichuan Science-observation Experimental Station of Veterinary Drugs and Veterinary Diagnostic Technology, Ministry of Agriculture, Chengdu, China

**Keywords:** *Haemophilus parasuis*, QseC, stress tolerance, biofilm formation, iron utilization

## Abstract

*Haemophilus parasuis* is known as a commensal organism discovered in the upper respiratory tract of swine where the pathogenic bacteria survive in various adverse environmental stress. QseC, a histidine protein kinase of the two-component regulatory systems CheY/QseC, is involved in the environmental adaptation in bacteria. To investigate the role of QseC in coping with the adverse environment stresses and survive in the host, we constructed a *qseC* mutant of *H. parasuis* serovar 13 strain (Δ*qseC*), MY1902. In this study, we found that QseC was involved in stress tolerance of *H. parasuis*, by the Δ*qseC* exhibited a decreased resistance to osmotic pressure, oxidative stress, and heat shock. Moreover, the Δ*qseC* weakened the ability to take up iron and biofilm formation. We also found that the QseC participate in sensing the epinephrine in environment to regulate the density of *H. parasuis*.

## Introduction

*Haemophilus parasuis* is a causative agent of *Glässer's* disease what characterized by fibrinous polyserositis, polyarthritis, and meningitis (Cai et al., 2005). *H. parasuis*, one of the major causes of nursery mortality in swine herds, giving rise to great economic loss in pig farms (Oliveira and Pijoan, 2004). After invading the respiratory tract and lung tissue, *H. parasuis* exposes to stress conditions, such as osmotic pressure, oxidative stress, and temperature. These stresses may affect bacterial survival, and result in protein denaturation and misfolding (Frees et al., 2004). *H. parasuis* regulates a variety of gene expression to survive these stress conditions (Huang et al., 2016). However, bacterial survival and establishment of infection require to sense and accurately to environmental cues. Two component regulatory systems are important for bacterial to adapt in the environment (Stock et al., 2000).

Two-component signal transduction system (TCSTS) consists of a sensor histidine kinase (HK) and a response regulator(RR) (Wuichet et al., 2010). QseBC, a two-component-based quorum sensing (QS) system that responds to hormone's signals AI-3, epinephrine, and norepinephrine in *Escherichia coli* and *Salmonella* (Sperandio et al., 2003; Clarke et al., 2006; Walters and Sperandio, 2006; Kalia, 2013). *Enterohemorrhagic E. coli* through QseC to senses AI-3, epinephrine and norepinephrine to activate flagella and motility, AE lesion formation and Shiga toxin expression (Hughes et al., 2009).

QseC sensory kinase is a bacterial adrenergic receptor that is crucial for interkingdom signaling in *E. coli* (Clarke et al., 2006). QseC, as a transmembrane protein with histidine protein kinase, is activated in response to host and bacterial signals, and phosphorylates the QseB response regulator, a transcription factor that regulates relevant virulence gene expression (Walters and Sperandio, 2006; Hughes et al., 2009)^1^. QseC can control QseB activation via a mechanism that is independent of reverse phosphotransfer. QseC-mediated dephosphorylation is required for maintaining proper QseB-PmrB-PmrA interactions in *Uropathogenic E. coli* (Breland et al., 2017). QseC controls biofilm formation in non-typeable *Haemophilus influenza* (Unal et al., 2012). Moreover, the Δ*qseC* diminished motility and colonization of the gastrointestinal tract compared to the wild-type parent strain in *Salmonella enterica* serovar Typhimurium (S. Typhimurium) (Bearson and Bearson, 2008). QseC also involved in flagellar motility, fimbrial hemagglutination, and intracellular virulence in fish pathogen *Edwardsiella tarda* (Wang et al., 2011).

TCSTS of bacteria are considered to form an intricate signal network to detect changes in environmental and respond by adjusting various cellular functions (Li et al., 2002; Eguchi et al., 2007)^2^^,^^3^. However, whether the QseC plays a role in the *H. parasuis* adapt to environmental changes in pigs are still unknown.

In the present study, we investigated the responses of Δ*qseC* to the stress conditions and biological characteristics which constructed by the natural transformation system (Bigas et al., 2005). In addition to affect biofilm formation as reported in *E. coli* and *Salmonella*, we found that QseC function in bacterial response to a variety of stimuli such as osmotic pressure, oxidative stress, and heat shock.

## Materials and methods

### Bacterial strains, plasmids, and growth conditions

The bacterial strains and plasmids used in this study were listed in Table 1. *E. coli* DH5a and *E. coli* BL21 were cultured in Luria–Bertani medium at 37°C. *H. parasuis* serovar 13 strain, MY1902 was grown in tryptic soy broth (TSB) medium or cultivated on tryptic soy agar (TSA) (Difco, Detroit, USA) supplemented with 0.001% (w/v) nicotinamide adenine dinucleotide (NAD) (Sigma Aldrich, Missouri, USA) and 5% (v/v) inactivated bovine serum at 37°C. When necessary, the media were supplemented with 50 μL kanamycin (100 mg/mL) or 100 μL ampicillin (100 mg/mL).

**Table 1:** Bacterial strains and plasmids used in this study.

**Strain or plasmid**	**Relevant characteristic(s)**	**Source**
**STRAIN**		
*H. parasuis* MY1902	serovar 13 clinical isolate	Laboratory collection
*H. parasuis* MY1902Δ*qseC*(Δ*qseC*)	MY1902Δ*qseC*:: Kan^r^	This study
*E. coli* DH5a	Cloning host for maintaining the recombinant plasmids	Tiangen
*E. coli* BL21	Expressing host for maintaining the recombinant plasmids	Tiangen
**PLASMID**		
pMD19-T	T-vector, Amp^r^	Takara
pET-32a	Expression vector, Amp^r^	Laboratory collection
pk18mobsacB	Suicide and narrow-broad-host vector, Kan^r^	Laboratory collection
pLQ2	A 2844-bp fragment containing Kan^r^, the upstream and downstream sequences of the *qseC* gene in pK18mobsacB, Kan^r^	This study
PLQ3	A 1601-bp fragment containing Gm^r^ and the *qseC* gene in PSF116	This study
PSF116	Gm resistance cassette-carrying vector, Gm^r^	Zhou et al., 2016
pLS88	Str^r^ resistance cassette-carrying complement vector, Str^r^ Kan^r^	Laboratory collection
pKD4	Amp^r^, Kan^r^, gene knock-out vector	Laboratory collection

*Kan, kanamycin; r, resistence*.

### Construction and complementation of the Δ*qseC* strain

The primers used to construct the *qseC* mutant are listed in Table 2. The 964-bp upstream and 945-bp downstream fragments of *qseC* were amplified from the genome of MY1902 using primers *qseC*-Up-F/R and *qseC*-Down-F/R, A kanamycin resistant (kanR) cassette(935 bp) was amplified from pKD4 using primers Kan-F/R. These three PCR fragments were combined by overlap PCR with primers *qseC*-up-F and *qseC*–down-R. Then the overlapped product was cloned into pK18mobsacB at BamHI and HindIII to construct the recombinant plasmid pLQ2. The recombinant plasmid pLQ2 was mobilized into *H. parasuis* strain MY1902 by natural transformations (Zhang et al., 2012b). To construct the complementing Plasmid pLQ3, the *qseC* gene was amplified from MY1902 using primers *qseC*-Comp-F/R and cloned into KpnI and BamHI digested pSF116 (Zhou et al., 2016). Both two DNA fragments (upstream homologous arm and down homologous arm) contained a 9-bp core DNA uptake signal sequence (USS) of 5′- ACCGCTTGT−3′ (Zhang et al., 2012a).

**Table 2:** Primers used in this study.

**Primer name**	**Sequence (5′–3′)**
*qseC*-Up-F	CGGGATCC**ACCGCTTGT**GCCAGCCAAGTATCTTCAATG (BamHI)
*qseC*-Up-R	ACTTTGCAGGGCTTCCCAACCTTACCGTTTTTTCCTAAGGCGTAGC
*qseC*-Comp-F	CGGGGTACCAGAGTACAATTTACTTGAGCTATTTATG (KpnI)
*qseC*-Comp-R	CGCGGATCCTCAGGACGGAGTTTGACGG (BamHI)
*qseC*-Down-F	ACTCTGGGGTTCGAAATGACCGACCAGGATGGAGATATAAGGCAC
*qseC*-Down-R	CCCAAGCTT**ACCGCTTGT**GTCTTTAGTGATGGTTGGTGC (HindIII)
*Kan*-F	GTAAGGTTGGGAAGCCCTGCAAAGT
*Kan*-R	GGTCGGTCATTTCGAACCCCAGAGT
*qseC*-F	CGGGATCCATGAAGTTGCTTAAAAATACC (*Bam*HI)
*qseC*-R	CCCAAGCTTTCAGGACGGAGTTTGACGGC (*Hin*dIII)
HPS-F	GTGATGAGGAAGGGTGGTGT
HPS-R	GGCTTCGTCACCCTCTGT

*Restriction sites are underlined, uptake signal sequences (USS) are in bold*.

### RT-PCR and western blotting

The RNAs of MY1902, Δ*qseC*, and C-Δ*qseC* were extracted using the Bacterial RNA Kit (5) according to the instructions(OMEGA R6950-00, America). RT-PCR using a PrimeScript™ RT reagent Kit (Perfect Real Time) according to the instructions (TaKaRa, Japan). The cDNA synthesis of both wild strain MY1902 and Δ*qseC* was detected with primers HPS-F/R, Kan-F/R, and *qseC*-F/R (Table 2). Western blotting assay was performed as described previously (Wang et al., 2013). 1 ml overnight bacterial cultures of the wild strain MY1902 and the Δ*qseC* were harvested by centrifugation for 1 min at 12000 rpm/min. Then resuspended with 40 μL ultrapure water, followed by adding 10 μL five-fold protein loading buffer, then boil for 10 min and ice bath for 2 min. The samples (10 ml) were electrophoresed on 12% SDS-PAGE gel and transferred onto Nitrocellulose(NC) membranes. The proteins on membrane were detected with Clarity™ Western ECL Substrate kit according to the instructions (BIO-RAD, America).

### Stress resistance assays

Stress resistance assays were performed as the previously described methods (Wong et al., 2007; Allen and Schmitt, 2009; Liu et al., 2013; Xie et al., 2013, 2016; Nasrallah et al., 2014; Huang et al., 2016). Fifty microliters of overnight cultures of MY1902, Δ*qseC*, and C-Δ*qseC* were subcultured at a dilution of 1:100 into 5 mL fresh TSB with 5% inactivated bovine serum and 0.01% NAD and the cells were grown at 37°C with 220 rpm. For the osmotic tolerance assay, the cells were cultivated on 40, 60, 80, and 100 mM NaCl TSA respectively. For the oxidative stress tolerance assay, the bacterial suspension was treated with 0.5, 1, 2, 4, 8, 16 mM H_2_O_2_ for 30 min respectively. For the heat-shock assay, bacterial cultures were placed in a 39, 42, and 45°C water bath for 30 min respectively and plated on TSA plates. Stress resistance was calculated as [(stressed sample CFU mL^−1^)/(control sample CFU mL^−1^)] × 100. The experiments were carried out independently three times.

### Iron utilization assays

Growth curve of the low-iron environment assay was performed as the previously described (Deslandes et al., 2007; Xie et al., 2013)^4^. Briefly, 1 mL overnight cultures of MY1902, Δ*qseC*, and C-Δ*qseC* were subcultured at a dilution of 1:100 into 100 mL fresh TSB with 100 μM, 200 μM EDDHA (ethylenediamine di(o-hydroxyphenylacetic) acid), a concentration sufficient to cause iron restriction (Beddek et al., 2004; Deslandes et al., 2007), or 33.33 μM FeSO4, 66.66 μM FeSO4 respectively, OD_600_ values were measured every hour. The experiments were carried out in triplicate independently.

### Biofilm formation assays

Biofilm formation ability was measured as described previously with some modifies (Kaplan and Mulks, 2005; Tremblay et al., 2013; Xie et al., 2016). Twenty microliters overnight cultures of MY1902, Δ*qseC*, and C-Δ*qseC* were subcultured at a dilution of 1:100 into fresh TSB in 6-well tissue culture plate for 24, 48, 72, 96 h at 37°C respectively. Biofilms were washed with water and stained with 1 ml of 0.1% crystal violet for30 min. Excess staining was rinsed off under water, drying, and 100 μL of 33% (v/v) acetic acid was added to each well, then transferred to 96-well polystyrenemicrotiter plates and measured at wavelength of 595 nm. All tests were repeated independently times.

### Confocal laser scanning microscopy

Confocal laser scanning microscopy (CLSM) assay was performed as previously described (Tremblay et al., 2013). Thirty microliters overnight cultures of MY1902, Δ*qseC*, and C-Δ*qseC* were subcultured were diluted 1:100 in the following media: fresh TSB, TSB with 50 μM epinephrine both supplemented with 5% inactivated bovine serum and 0.01% NAD in the six well microtiter plate with 20 × 20 mm cell climbing tablets for 18 h. The cell climbing tablets were removed and washed three times with phosphate buffer saline and stained with (LIVE/DEAD^@^ BacLight ™ Bacterial Viability kits, Invitrogen; live bacteria stain fluorescent green, whereas dead bacteria stain fluorescent red). The plates were incubated at room temperature in the dark for 20 min and washed three times with phosphate buffer saline. SYTO 9 was excitation (with an Ar laser) at 488 nm, and propidium iodide was excitation (with a HeNe laser) at 559 nm using Nikon AIR confocal scanning laser microscope (CLSM). The images were analyzed with the NIS-Elements AR software.

## Results

### Sequence analysis of QseC protein

**Figure 1:**
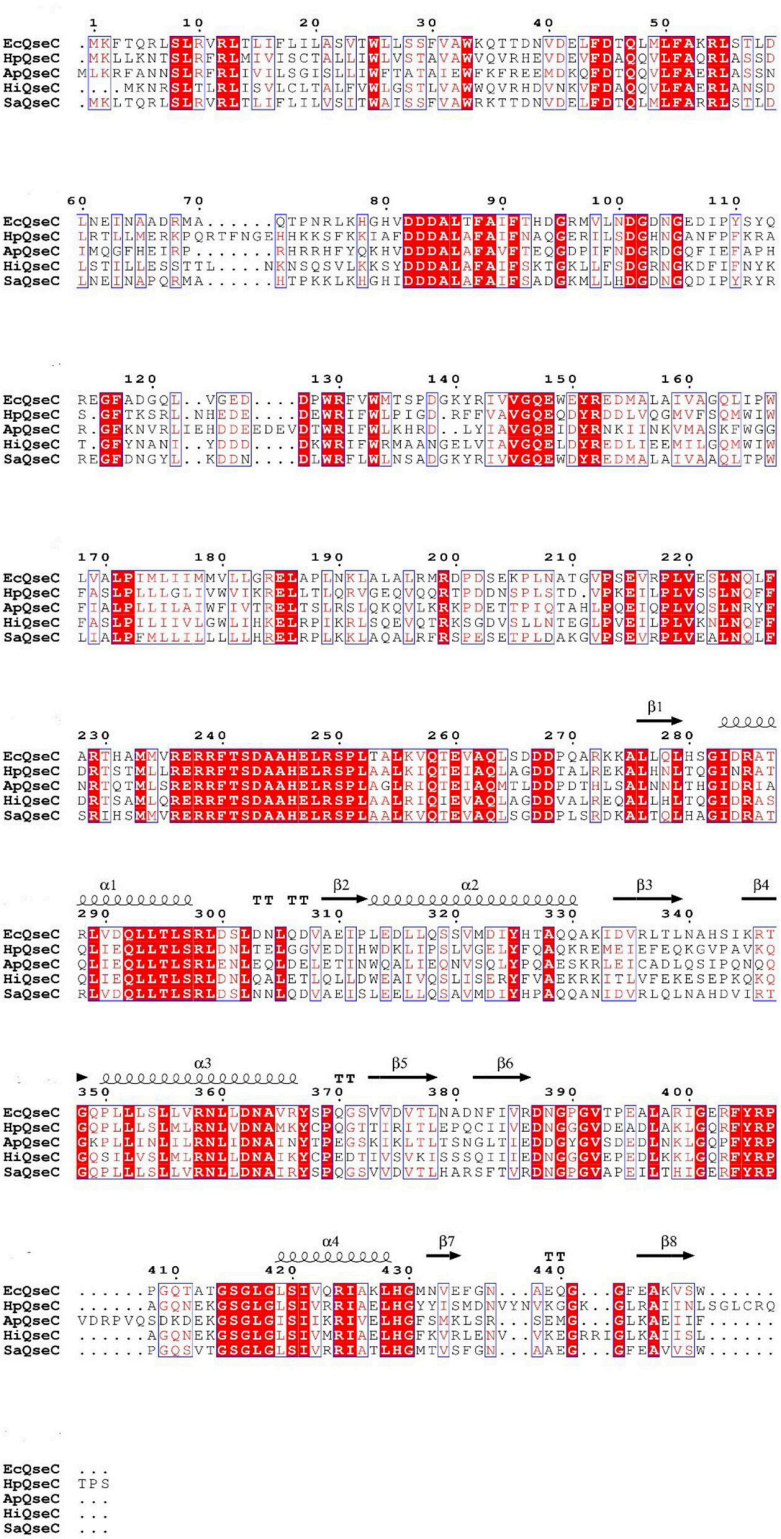
Sequence alignment of *H. parasuis* QseC (HpQseC) against *A. pleuropneumoniae* QseC(ApQseC), *H. influenzae* QseC (HiQseC), *Salmonella* QseC (SaQseC), and *E. coli* QseC (EcQseC).

QseC is a chromosomally encoded polypeptide with 466 amino acids. In order to determine the similarity between QseC, we aligned the protein sequences of QseC from *H. parasuis* (SH0165) with *Actinobacillus pleuropneumoniae* (L20), *H. influenzae* (Rd KW20), *S. enterica subsp* (enterica serovar Typhimurium str. LT2), and *E. coli* (str. K-12 substr. MG1655). The multiple-sequence alignment revealed 60.71% identity (Figure 1).

### Construction and complementation of the *qseC* knockout mutant in *H. parasuis*

In this study, we constructed the *qseC* mutant (Δ*qseC*) and complementary strain (C-Δ*qseC*) of *H. parasuis* serovar 13 strain, MY1902 (Figure 2 in Supplementary Material). And the result of reverse transcription PCR(RT-PCR) verificated the *H. parasuis* Δ*qseC* was successfully constructed (Figure 3 in Supplementary Material). Additionally, the result of western blot showed that the expression of a protein of ~69.08 kDa was absent in the Δ*qseC* compared with the wild-type strain MY1902 (Figure 4 in Supplementary Material). These results indicated that the *qseC* gene have been knocked out from the genome of the wild-type strain MY1902.

### Growth assays

**Figure 2:**
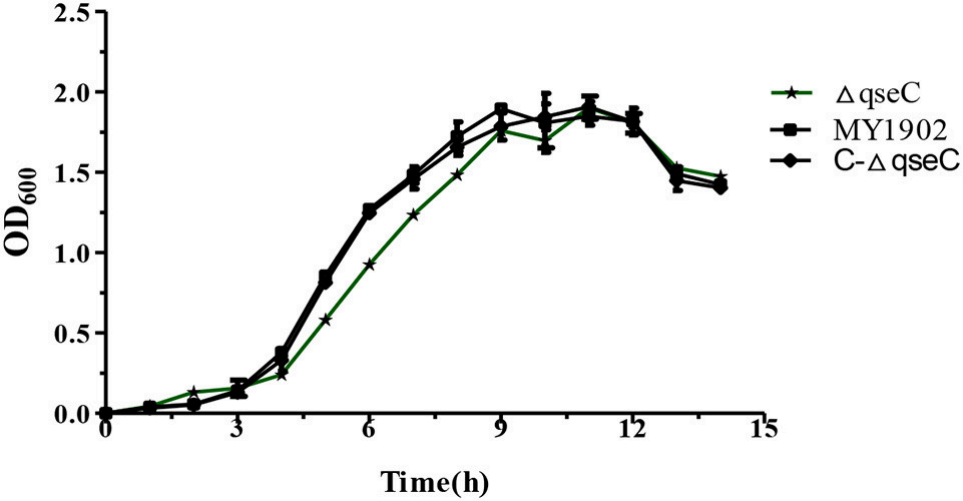
Growth of the wild strain MY1902, Δ*qseC* and C-Δ*qseC* in TSB supplemented with 5% inactivated bovine serum and 0.01% NAD. Error bars represent the standard deviations of three independent experiments.

Compared with the wild strain MY1902, we found that the Δ*qseC* didn't exhibit obvious growth defects compared with the wild strain (Figure 2).

### Loss of QseC showed more sensitive to stress conditions

**Figure 3:**
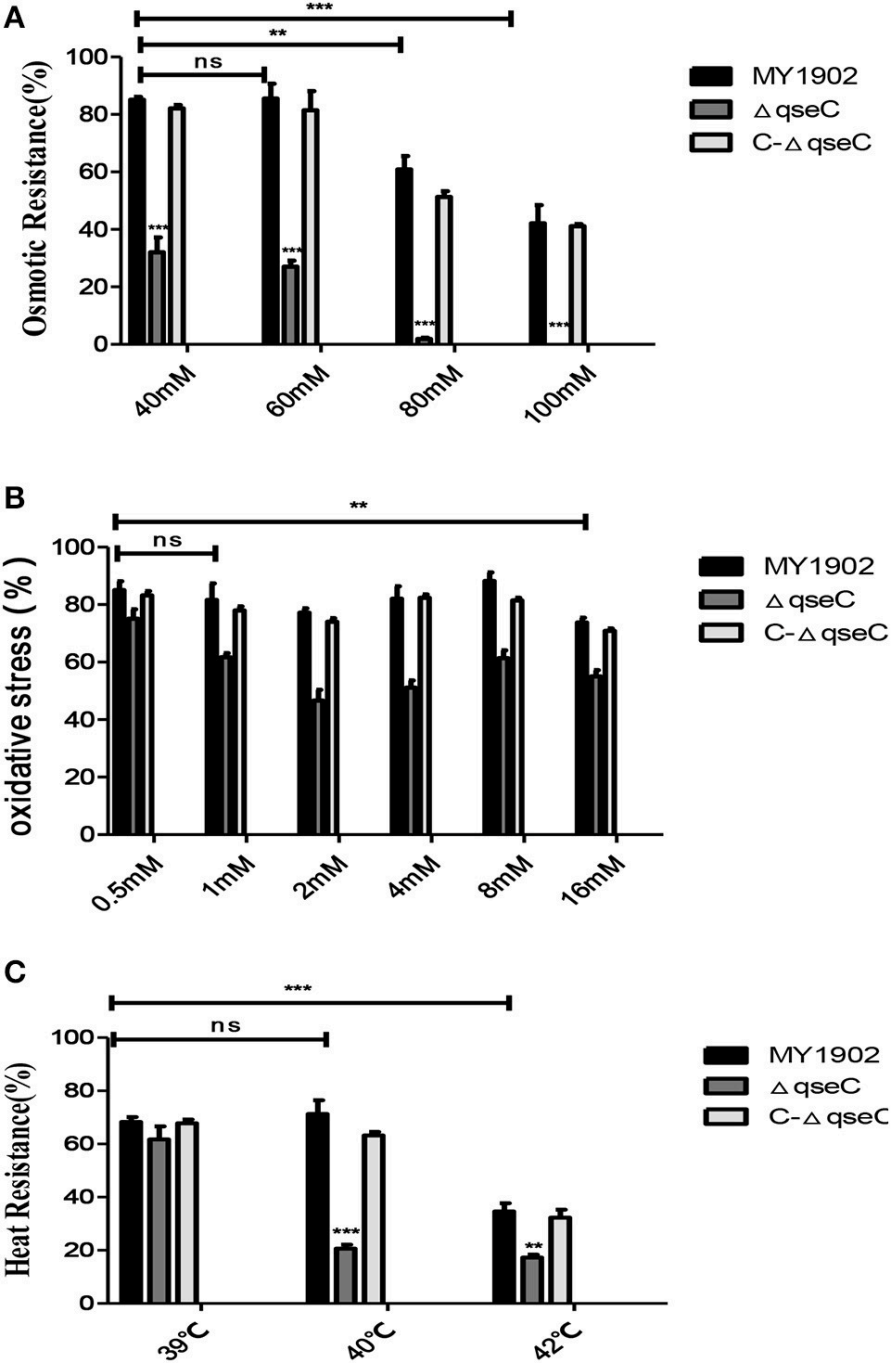
Analysis of the stress tolerance of wild strain MY1902, Δ*qseC* and C-Δ*qseC*. **(A)** The bacterial were treated with 40, 60, 80, and 100 mM NaCl TSA, **(B)** The bacterial exposed to 0.5, 1, 2, 4, 8, 16 mM H_2_O_2_ for 30 min, **(C)** The bacterial were incubated in 39, 42, and 45°C water bath for 30 min. Data indicate the mean of three independent experiments performed in duplicates and error bars show SDs. Asterisks indicate statistical significance using two-way ANOVA (^**^P < 0.01; ^***^P < 0.001).

We investigated various stress conditions included osmotic pressure, oxidative stress, and heat shock of the wild strain, Δ*qseC* and C-Δ*qseC*. When bacterial were treated with 40, 60, 80, and 100 mM NaCl TSA, the survival rate of Δ*qseC* was 32, 27.07, 1.88%, 0, which much lower than the wild strain with 85.13, 87.22, 60.88, 42.1% survival (Figure 3A). Similar results were observed in the oxidative stress assay, when bacterial exposed to 0.5, 1, 2, 4, 8, 16 mM H_2_O_2_ for 30 min, the survival rate of wild strain were 85.04, 81.64, 77.24, 82.07, 88.24, 73.8%, while only 75.13, 61.73, 46.6, 51.13, 61.38, 55.01% of the Δ*qseC* cells survived (Figure 3B). When incubated in 39, 42 and 45°C water bath for 30 min, the wild strain exhibited survival rates of 68.25, 71.30, 34.6%. However, the resistance of Δ*qseC* to heat-shock was significantly decreased, with survival rate of 61.74, 20.59, and 17.30% (Figure 3C). These findings indicated that QseC played an important role in stress tolerance in *H. parasuis*.

### The QseC influenced iron utilization of *H. parasuis*

**Figure 4:**
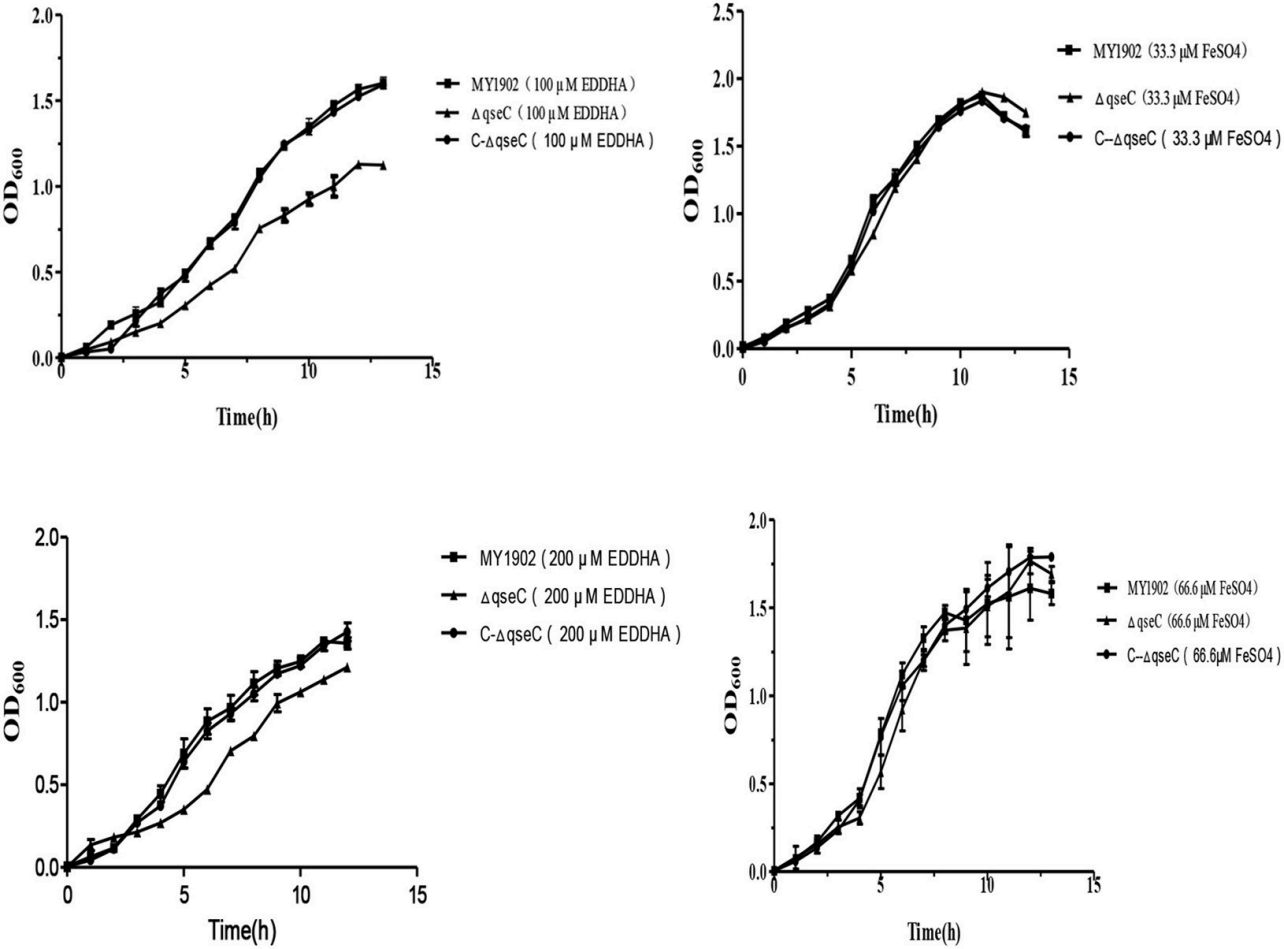
The capability of utilize iron of the wild strain MY1902, Δ*qseC*, and C-Δ*qseC*. Growth of the wild strain MY1902, Δ*qseC* and C-Δ*qseC* in iron restricted medium and iron supplementation medium. Error bars represent the standard deviations of three independent experiments.

The ability of utilize iron in the wild strain MY1902 and the *qseC* mutant strain were studied by using of iron restricted medium with 100, 200 μM EDDHA or 33.33 μM FeSO4, 66.66 μM FeSO4 respectively, containing 5% inactivated bovine serum and 0.01%NAD. As shown in Figure 3, when exposed to 100 /200 μM EDDHA, the Δ*qseC* decreased the growth compared with the wild strain in *H. parasui*s. Whereas supplement of FeSO4, the Δ*qseC* restored the growth rate (Figure 4).

### The Δ*qseC* showed impaired biofilm formation

**Figure 5:**
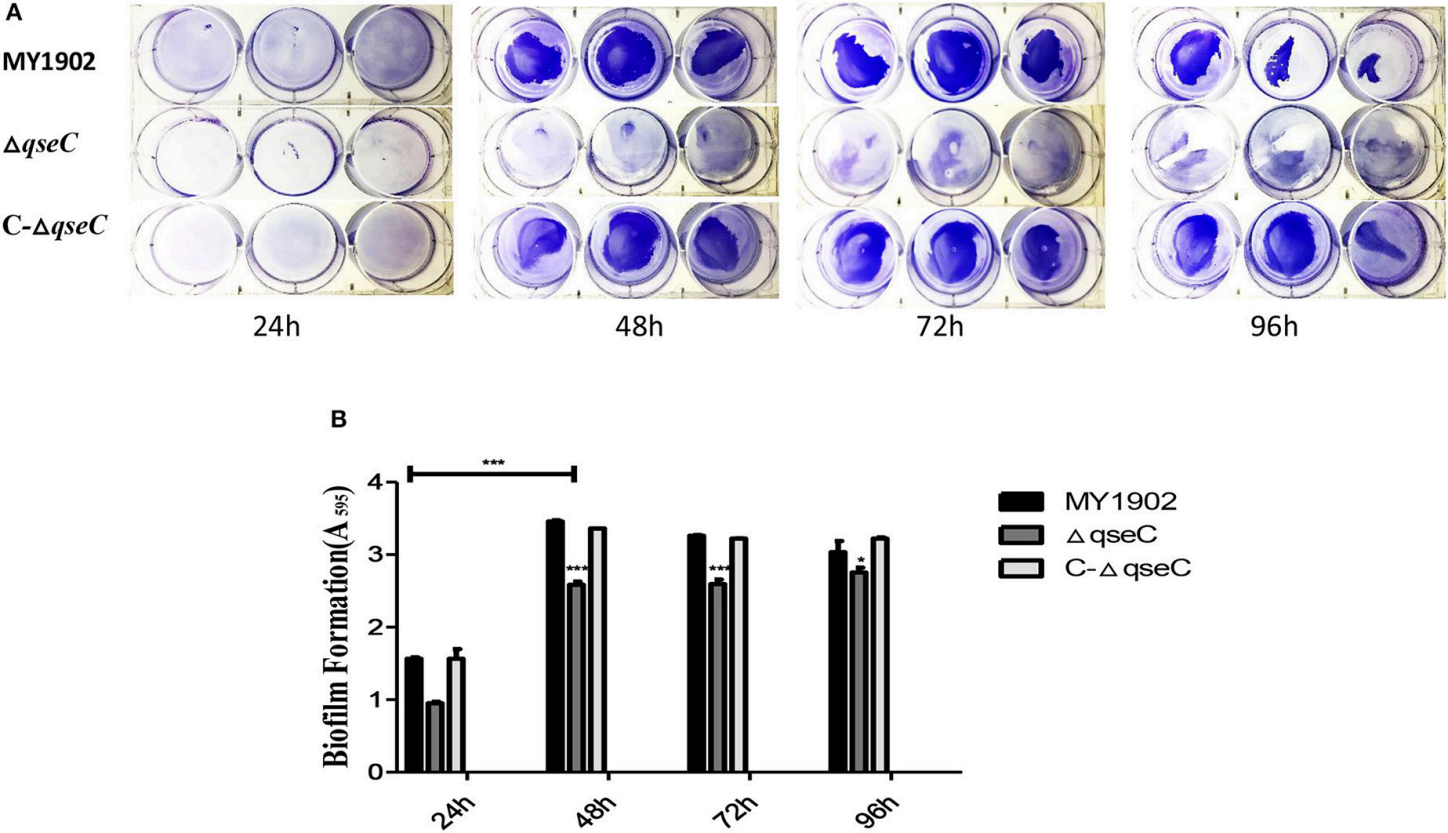
**(A)** Biofilms were stained with crystal violet. **(B)** Quantification of biofilm productions. Error bars represent the standard deviations of three independent experiments. Asterisks indicate statistical significance using two-way ANOVA (^*^P < 0.05; ^***^P < 0.001).

The biofilm formation assay results show that QseC was involved in biofilm formation in *H. parasuis* (Figure 5). Biofilm productions was measured at wavelength of 595 nm.

### The QseC might sense the epinephrine in environment to regulate the density of *H. parasuis*

**Figure 6:**
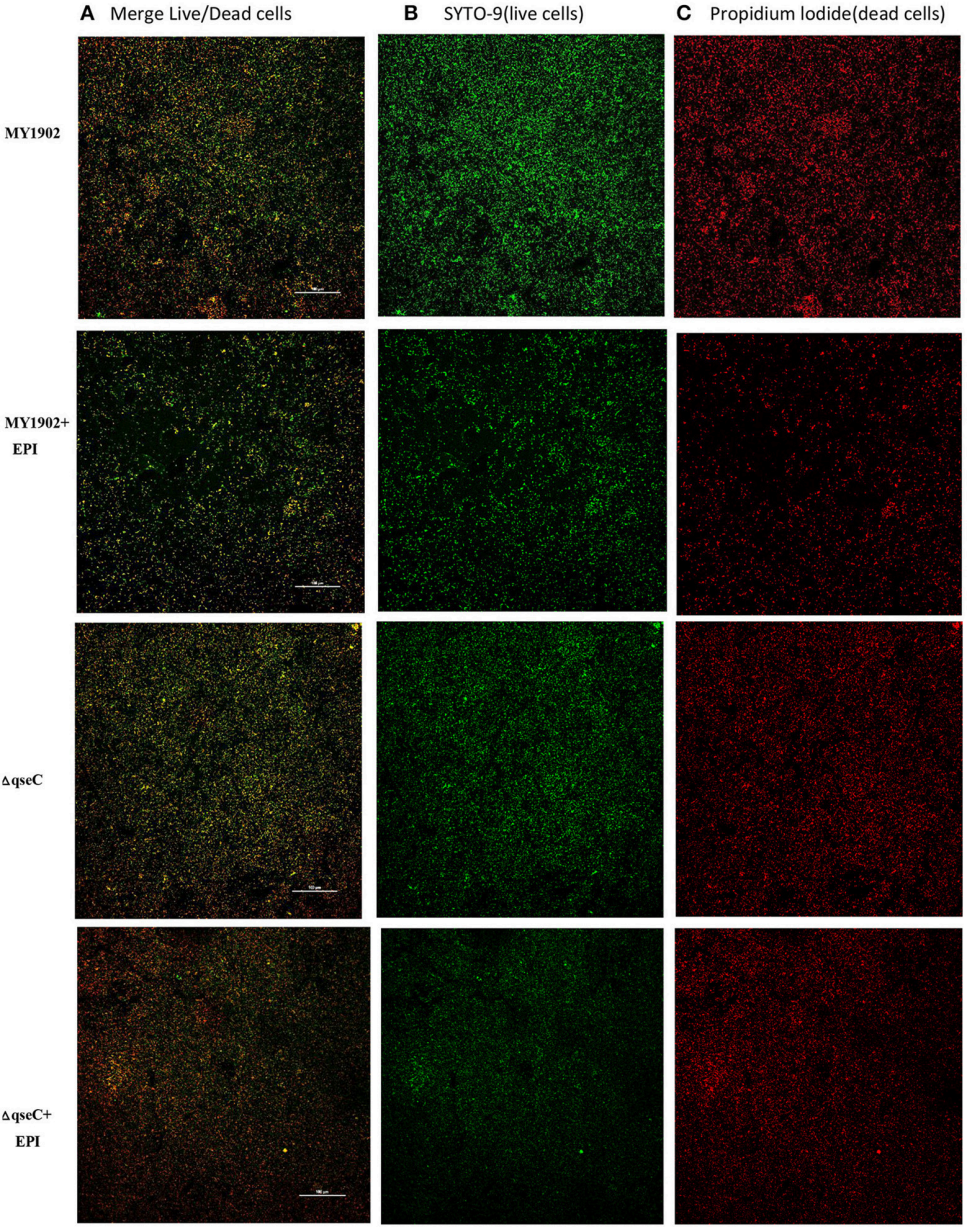
CLSM images of MY1902 and Δ*qseC. H. parasuis* were cultured in six-well microtiter plates were stained with the SYTO-9 and propidium iodide to label live versus dead cells.

Previously study proved that *qseBC* is activated by AI-3. AI-3, and Epi are recognized by the same receptor, and Δ*qseC* is unable to respond to both AI-3 and Epi in *Enterohemorrhagic E. coli* (Sperandio et al., 2003). We found that when exposed to 50 μM exogenous epinephrine, the Δ*qseC* weakened the ability of feeling the signal AI-3 (Figure 6). The specific mechanisms of interkingdom communication still unkown, and further research are needed in this specific mechanisms, which is important in understanding bacterial pathogenesis.

## Discussion

Bacteria have multiple mechanisms for sensing the environment and regulate gene expressions in response to different niches of their host organisms, which is frequently mediated by TCSTS (Labandeira-Rey et al., 2010; Xu et al., 2014). CheY/QseC is one of the TCSTS in *H. parasuis*, and involved in virulence gene expression in a number of pathogenic bacterias (Kostakioti et al., 2009). QseC controls the phosphorylation of QseB in order to optimize expression patterns (metabolic and virulence genes) in *E. coli* (Hadjifrangiskou et al., 2011). QseBC plays an important role in flagellar motility, fimbrial hemagglutination, and intracellular virulence in fish pathogen Edwardsiella tarda (Wang et al., 2011). QseBC of *E. coli* shares the homology with *H. parasuis* regulator Y and C (CheY/QseC). However, the functions of QseC in the *H. parasuis* adapts to the environment are unknown. It is necessary to explore the function of the QseC in *H. parasuis*.

In this study, we constructed a *qseC* deletion mutant of *H. parasuis* serovar 13 strain MY1902(Δ*qseC*), and studied the survival rate under a variety of stress conditions as well as relevant biological characteristics. Results showed that the QseC played an important role in stress tolerance and biofilm formation of *H. parasuis* strain. Whereas we found that the Δ*qseC* didn't exhibit obvious growth defects compared with the wild strain. In this study, we observed that the Δ*qseC* was more sensitive to osmotic pressure, oxidative stress, and heat shock. Most notably the Δ*qseC* significantly osmotic pressure tolerance, in which the survival rate of wild strain was 42.1% whereas the Δ*qseC* didn't grow when exposed to 100 mM NaCl. These data suggested that the QseBC two-component system participated in *H. parasuis* responded to signal in the environment and survived in the stress conditions.

Iron is essential for bacterial growth, and it's an environmental signal that regulates the expression of virulence factors (Jacques, 2004). Iron contributes a lot to the growth of *H. parasuis*, and low iron availability in the host is a primary pressure for the pathogenic bacterium and considered a signal that leads to significant changes in cell processes (Deslandes et al., 2007). In this study we found that QseC might regulate the expression of some of the genes involved in iron uptake, and further studies will be necessary to evaluate the impact of the two-component regulatory systems CheY/QseC during the course of iron acquisition in *H. parasuis*.

Furthermore, we observed that the biofilm formation ability of the Δ*qseC* was weaker than the wild-type strain. Bacterial biofilm formation is a complex multifactor process, which is involved in adherence, competence, quorum sensing, cell wall synthesis, metabolism, and the stress response (Hasona et al., 2007). Previous studies have demonstrated that *qseC* controls biofilm formation of non-typeable *H. influenza* (Unal et al., 2012). Similarly, protein CheY was proved to influence biofilm formation in *H. parasui*s (He et al., 2016), which might closely related with QseC. In this study, we demonstrated that QseC was involved in biofilms formation in *H. parasuis*.

QseC, a histidine sensor kinase that can sense epinephrine (EPI)/norepinephrine (NE) was the quorum-sensing regulator of *E. coli and Eschericha coli* and *S. enterica* (Yang et al., 2014; Weigel et al., 2015). Weigel et al. demonstrated that iron and catecholamines may be signals that activate the QseC sensor, and detection of catecholamines and iron by the QseBC two-component system may essential for the adaptation of *A. actinomycetemcomitans* to the host cell environment (Weigel et al., 2015). Privious study demonstrated that epinephrine (EPI) and norepinephrine (NE) can promote the growth of a variety of bacteria, including *Pseudomonas aeruginosa, Yersinia enterocolitica, E. coli* (Green et al., 2003). But some scholars noted that the addition of 50 μM EPI or NE did not accelerate *E. coli* growth, which was in contrast to prior reports describing that NE increased the growth of *E. coli* and other bacteria (Yang et al., 2014). Interestingly, we found that the addition of 50 μM epinephrine reduced the cell density of MY1902 in *H. parasuis*, which might had an relationship with the QseC quorum-sensing sensor kinase. The Δ*qseC*, which lacks the ability to sense the hormones, showed little difference when exposure to epinephrine in the environment. Detection of AI-3 by the QseBC two-component system may play an important role in the growth of *H. parasuis*.

In conclusion, we successfully constructed the Δ*qseC*, C-Δ*qseC* and investigated the functions of QseC in the *H. parasuis* on stress response, iron utilization, biofilm formation and sense epinephrine. The Δ*qseC* obviously weakened the ability of stress tolerance such as osmotic pressure, oxidative stress, heat shock. In addition, the Δ*qseC* decreased the ability of iron acquisition and biofilm formation compared with the wild-type strain MY1902 in *H. parasuis*, which suggested that QseBC two-component system played an important role in sensing the external stimuli and adapt to environmental pressures. Further studies are needed to determine the regulatory mechanism of transmembrane protein QseC interacted with the response regulator CheY in *H. parasuis*.

## Author contributions

XW, SC, XHu, RW, YH, and QZ: designed this experiment; LH, KD, LD, and YW: implement the experimental program the experimental program; QY, XM, and XHa: modify the articles; LH: organize data and write articles.

### Conflict of interest statement

The authors declare that the research was conducted in the absence of any commercial or financial relationships that could be construed as a potential conflict of interest.

## Footnotes

^1^Quorum sensing Escherichia coli regulators B and C (QseBC)_ a novel two-component regulatory systeminvolved in the regulation of flagella and motility byquorum sensing in *E. coli*.pdf.

^2^Signaling by two-component system non-cognate partners promotes intrinsic tolerance to polymyxin B in uropathogenic *Escherichia coli*.pdf.

^3^Strong cross-system interactions drive the activation of the Qse B response regulator in the absence of its cognate sensor.pdf.

^4^Molecular cloning and characterization of the ferric hydroxamate uptake (fhu) operon in *Actinobacillus pleuropneumoniae*.pdf.

## References

[B1] Allen C. E. Schmitt M. P. (2009). HtaA is an iron-regulated hemin binding protein involved in the utilization of heme iron in *Corynebacterium diphtheriae*. J. Bacteriol. 191, 2638–2648. 10.1128/JB.01784-08 19201805 PMC2668399

[B2] Bearson B. L. Bearson S. M. (2008). The role of the QseC quorum-sensing sensor kinase in colonization and norepinephrine-enhanced motility of *Salmonella enterica* serovar Typhimurium. Microb. Pathog. 44, 271–278. 10.1016/j.micpath.2007.10.001 17997077

[B3] Beddek A. J. Sheehan B. J. Bossé J. T. Rycroft A. N. Kroll J. S. Langford P. R. (2004). Two TonB systems in *Actinobacillus pleuropneumoniae*: their roles in iron acquisition and virulence. Infect. Immun. 72, 701–708. 10.1128/IAI.72.2.701-708.2004 14742511 PMC321588

[B4] Bigas A. Garrido M. E. de Rozas A. M. Badiola I. Barbé J. Llagostera M. (2005). Development of a genetic manipulation system for *Haemophilus parasuis*. Vet. Microbiol. 105, 223–228. 10.1016/j.vetmic.2004.10.015 15708819

[B5] Breland E. J. Zhang E. W. Bermudez T. Martinez C. R. III. Hadjifrangiskou M. (2017). The histidine residue of QseC is required for canonical signaling between QseB and PmrB in uropathogenic *Escherichia coli*. J. Bacteriol. 199:e00060–17. 10.1128/JB.00060-17 28396353 PMC5573081

[B6] Cai X. Chen H. Blackall P. J. Yin Z. Wang L. Liu Z.. (2005). Serological characterization of *Haemophilus parasuis* isolates from China. Vet. Microbiol. 111, 231–236. 10.1016/j.vetmic.2005.07.007 16271834

[B7] Clarke M. B. Hughes D. T. Zhu C. Boedeker E. C. Sperandio V. (2006). The QseC sensor kinase: a bacterial adrenergic receptor. Proc. Natl. Acad. Sci. U.S.A. 103, 10420–10425. 10.1073/pnas.0604343103 16803956 PMC1482837

[B8] Deslandes V. Nash J. H. Harel J. Coulton J. W. Jacques M. (2007). Transcriptional profiling of *Actinobacillus pleuropneumoniae* under iron-restricted conditions. BMC Genomics 8:72. 10.1186/1471-2164-8-72 17355629 PMC1832192

[B9] Eguchi Y. Itou J. Yamane M. Demizu R. Yamato F. Okada A.. (2007). B1500, a small membrane protein, connects the two-component systems EvgS/EvgA and PhoQ/PhoP in *Escherichia coli*. Proc. Natl. Acad. Sci. U.S.A. 104, 18712–18717. 10.1073/pnas.0705768104 17998538 PMC2141842

[B10] Frees D. Chastanet A. Qazi S. Sørensen K. Hill P. Msadek T.. (2004). Clp ATPases are required for stress tolerance, intracellular replication and biofilm formation in *Staphylococcus aureus*. Mol. Microbiol. 54, 1445–1462. 10.1111/j.1365-2958.2004.04368.x 15554981

[B11] Green B. T. Lyte M. Kulkarni-Narla A. Brown D. R. (2003). Neuromodulation of enteropathogen internalization in Peyer's patches from porcine jejunum. J. Neuroimmunol. 141, 74–82. 10.1016/S0165-5728(03)00225-X 12965256

[B12] Hadjifrangiskou M. Kostakioti M. Chen S. L. Henderson J. P. Greene S. E. Hultgren S. J. (2011). A central metabolic circuit controlled by QseC in pathogenic *Escherichia coli*. Mol. Microbiol. 80, 1516–1529. 10.1111/j.1365-2958.2011.07660.x 21542868 PMC3643513

[B13] Hasona A. Zuobi-Hasona K. Crowley P. J. Abranches J. Ruelf M. A. Bleiweis A. S.. (2007). Membrane composition changes and physiological adaptation by *Streptococcus mutans* signal recognition particle pathway mutants. J. Bacteriol. 189, 1219–1230. 10.1128/JB.01146-06 17085548 PMC1797365

[B14] He L. Wen X. Yan X. Ding L. Cao S. Huang X.. (2016). Effect of cheY deletion on growth and colonization in a *Haemophilus parasuis* serovar 13 clinical strain EP3. Gene 577, 96–100. 10.1016/j.gene.2015.11.046 26657038

[B15] Huang J. Wang X. Cao Q. Feng F. Xu X. Cai X. (2016). ClpP participates in stress tolerance and negatively regulates biofilm formation in *Haemophilus parasuis*. Vet. Microbiol. 182, 141–149. 10.1016/j.vetmic.2015.11.020 26711041

[B16] Hughes D. T. Clarke M. B. Yamamoto K. Rasko D. A. Sperandio V. (2009). The QseC adrenergic signaling cascade in *Enterohemorrhagic E. coli* (EHEC). PLoS Pathog. 5:e1000553. 10.1371/journal.ppat.1000553 19696934 PMC2726761

[B17] Jacques M. (2004). Surface polysaccharides and iron-uptake systems of *Actinobacillus pleuropneumoniae*. Can. J. Vet. Res. 68, 81–85. 15188950 PMC1142149

[B18] Kalia V. C. (2013). Quorum sensing inhibitors: an overview. Biotechnol. Adv. 31, 224–245. 10.1016/j.biotechadv.2012.10.004 23142623

[B19] Kaplan J. B. Mulks M. H. (2005). Biofilm formation is prevalent among field isolates of *Actinobacillus pleuropneumoniae*. Vet. Microbiol. 108, 89–94. 10.1016/j.vetmic.2005.02.011 15917136

[B20] Kostakioti M. Hadjifrangiskou M. Pinkner J. S. Hultgren S. J. (2009). QseC-mediated dephosphorylation of QseB is required for expression of genes associated with virulence in uropathogenic *Escherichia coli*. Mol. Microbiol. 73, 1020–1031. 10.1111/j.1365-2958.2009.06826.x 19703104 PMC2963169

[B21] Labandeira-Rey M. Brautigam C. A. Hansen E. J. (2010). Characterization of the CpxRA regulon in *Haemophilus ducreyi*. Infect. Immun. 78, 4779–4791. 10.1128/IAI.00678-10 20805330 PMC2976327

[B22] Li Y. H. P. Lau C. Y. Tang N. Svensäter G. Ellen R. P. Cvitkovitch D. G. (2002). Novel two-component regulatory system involved in biofilm formation and acid resistance in *Streptococcus mutans*. J. Bacteriol. 184, 6333–6342. 10.1128/JB.184.22.6333-6342.2002 12399503 PMC151940

[B23] Liu M. Bouhsira E. Boulouis H. J. Biville F. (2013). The Bartonella henselae SitABCD transporter is required for confronting oxidative stress during cell and flea invasion. Res. Microbiol. 164, 827–837. 10.1016/j.resmic.2013.06.009 23811032

[B24] Nasrallah G. K. Abdelhady H. Tompkins N. P. Carson K. R. Garduño R. A. (2014). Deletion of potD, encoding a putative spermidine-binding protein, results in a complex phenotype in *Legionella pneumophila*. Int. J. Med. Microbiol. 304, 703–716. 10.1016/j.ijmm.2014.05.004 24928741

[B25] Oliveira S. Pijoan C. (2004). *Haemophilus parasuis*: new trends on diagnosis, epidemiology and control. Vet. Microbiol. 99, 1–12. 10.1016/j.vetmic.2003.12.001 15019107

[B26] Sperandio V. Torres A. G. Jarvis B. Nataro J. P. Kaper J. B. (2003). Bacteria-host communication: the language of hormones. Proc. Natl. Acad. Sci. U.S.A. 100, 8951–8956. 10.1073/pnas.1537100100 12847292 PMC166419

[B27] Stock A. M. Robinson V. L. Goudreau P. N. (2000). Two-component signal transduction. Annu. Rev. Biochem. 69, 183–215. 10.1146/annurev.biochem.69.1.183 10966457

[B28] Tremblay Y. D. Deslandes V. Jacques M. (2013). *Actinobacillus pleuropneumoniae* genes expression in biofilms cultured under static conditions and in a drip-flow apparatus. BMC Genomics 14:364. 10.1186/1471-2164-14-364 23725589 PMC3671958

[B29] Unal C. M. Singh B. Fleury C. Singh K. L. Chávez de Paz Svensáter G.. (2012). QseC controls biofilm formation of non-typeable *Haemophilus influenzae* in addition to an AI-2-dependent mechanism. Int. J. Med. Microbiol. 302, 261–269. 10.1016/j.ijmm.2012.07.013 22954413

[B30] Walters M. Sperandio V. (2006). Quorum sensing in *Escherichia coli* and Salmonella. Int. J. Med. Microbiol. 296, 125–131. 10.1016/j.ijmm.2006.01.041 16487745

[B31] Wang X. Wang Q. Yang M. Xiao J. Liu Q. Wu H.. (2011). QseBC controls flagellar motility, fimbrial hemagglutination and intracellular virulence in fish pathogen *Edwardsiella tarda*. Fish Shellfish Immunol. 30, 944–953. 10.1016/j.fsi.2011.01.019 21288493

[B32] Wang X. Xu X. Wu Y. Li L. Cao R. Cai X.. (2013). Polysaccharide biosynthesis protein CapD is a novel pathogenicity-associated determinant of *Haemophilus parasuis* involved in serum-resistance ability. Vet. Microbiol. 164, 184–189. 10.1016/j.vetmic.2013.01.037 23434184

[B33] Weigel W. A. Demuth D. R. Torres-Escobar A. Juárez-Rodríguez M. D. (2015). *Aggregatibacter actinomycetemcomitans* QseBC is activated by catecholamines and iron and regulates genes encoding proteins associated with anaerobic respiration and metabolism. Mol. Oral Microbiol. 30, 384–398. 10.1111/omi.12101 25923132 PMC4660874

[B34] Wong S. M. Alugupalli K. R. Ram S. Akerley B. J. (2007). The ArcA regulon and oxidative stress resistance in *Haemophilus influenzae*. Mol. Microbiol. 64, 1375–1390. 10.1111/j.1365-2958.2007.05747.x 17542927 PMC1974803

[B35] Wuichet K. Cantwell B. J. Zhulin I. B. (2010). Evolution and phyletic distribution of two-component signal transduction systems. Curr. Opin. Microbiol. 13, 219–225. 10.1016/j.mib.2009.12.011 20133179 PMC3391504

[B36] Xie F. Li G. Zhang Y. Zhou L. Liu S. Liu S. (2016). The Lon protease homologue LonA, not LonC, contributes to the stress tolerance and biofilm formation of *Actinobacillus pleuropneumoniae*. Microb. Pathog. 93, 38–43. 10.1016/j.micpath.2016.01.009 26796296

[B37] Xie F. Zhang Y. Li G. Zhou L. Liu S. Wang C. (2013). The ClpP protease is required for the stress tolerance and biofilm formation in *Actinobacillus pleuropneumoniae*. PLoS ONE 8:e53600. 10.1371/journal.pone.0053600 23326465 PMC3543445

[B38] Xu J. Fu S. Liu M. Xu Q. Bei W. Chen H.. (2014). The two-component system NisK/NisR contributes to the virulence of *Streptococcus suis* serotype 2. Microbiol. Res. 169, 541–546. 10.1016/j.micres.2013.11.002 24342108

[B39] Yang K. Meng J. Huang Y. C. Ye L. H. Li G. J. Huang J.. (2014). The role of the QseC quorum-sensing sensor kinase in epinephrine-enhanced motility and biofilm formation by *Escherichia coli*. Cell Biochem. Biophys. 70, 391–398. 10.1007/s12013-014-9924-5 24676679

[B40] Zhang B. Feng S. Xu C. Zhou S. He Y. Zhang L.. (2012a). Serum resistance in *Haemophilus parasuis* SC096 strain requires outer membrane protein P2 expression. FEMS Microbiol. Lett. 326, 109–115. 10.1111/j.1574-6968.2011.02433.x 22092746

[B41] Zhang B. He Y. Xu C. Xu L. Feng S. Liao M.. (2012b). Cytolethal distending toxin (CDT) of the *Haemophilus parasuis* SC096 strain contributes to serum resistance and adherence to and invasion of PK-15 and PUVEC cells. Vet. Microbiol. 157, 237–242. 10.1016/j.vetmic.2011.12.002 22221379

[B42] Zhou Q. Feng S. Zhang J. Jia A. Yang K. Xing K.. (2016). Two glycosyltransferase genes of *Haemophilus parasuis* SC096 implicated in lipooligosaccharide biosynthesis, serum resistance, adherence, and invasion. Front. Cell. Infect. Microbiol. 6:100. 10.3389/fcimb.2016.00100 27672622 PMC5018477

