# Supplementary material for: QseC Mediates Osmotic Stress Resistance and Biofilm Formation in Haemophilus parasuis
Source: Front Microbiol. 2018 Feb 13;9:212. doi: 10.3389/fmicb.2018.00212 (PMC5816903; doi:10.3389/fmicb.2018.00212)
Supplement: Supplementary file 1 [file DataSheet1.docx]

**Annex 1**

**M 1 2 3 4 5 6 7 8 9**


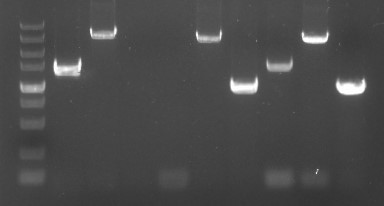


**5000bp→**

**3000bp→**

**2000bp→**

**1500bp→**

**1000bp→**

**750bp→**

**500bp→**

**250bp→**

**100bp→**

Fig. 2. PCR identification of the wild strain, △*qseC* and C-△*qseC*. Lane 1: PCR amplification by *qseC*-F/R from MY1902 strain; Lane 2: PCR amplification by *qseC* upstream to downstream from MY1902 strain; Lane 3: PCR amplification by Kan-F/R from MY1902 strain; Lane 4: PCR amplification by *qseC*-F/R from the △*qseC* strain; Lane5: PCR amplification by *qseC* upstream to downstream from the △*qseC* strain; Lane6: PCR amplification by Kan-F/R from the △*qseC*. Lane7: PCR amplification by *qseC*-F/R from C-△*qseC*; Lane8: PCR amplification by *qseC* upstream to downstream from C-△*qseC*; Lane9: PCR amplification by Kan-F/R from C-△*qseC*. Lane M shows a DNA molecular marker.

**M 1 2 3 4 5 6 7 8 9**


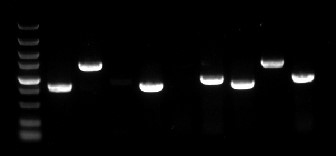


**5000bp→**

**3000bp→**

**2000bp→**

**1500bp→**

**1000bp→**

**750bp→**

**500bp→**

**250bp→**

**100bp→**

Fig. 3. reverse transcription PCR(RT-PCR) verification of the wild strain,△*qseC* and C-△*qseC*. Lane 1: PCR amplification by HPS-F/R from MY1902 strain; Lane 2: PCR amplification by *qseC-*F/R from MY1902 strain; Lane 3: PCR amplification by Kan-F/R from MY1902 strain; Lane 4: PCR amplification by HPS-F/R from the △*qseC* strain; Lane5: PCR amplification by *qseC-*F/R from the △*qseC* strain; Lane6: PCR amplification by Kan-F/R from the△*qseC* strain. Lane7: PCR amplification by HPS-F/R from C-△*qseC*; Lane8: PCR amplification by *qseC-*F/R from the C-△*qseC*; Lane9: PCR amplification by Kan-F/R from the C-△*qseC*. Lane M shows a DNA molecular marker.


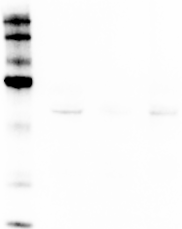


←

**250KD→**

**150KD→**

**100KD→**

**75KD→**

**50 KD→**

**25 KD→**

**10 KD→**

Fig. 4. Western blotting analysis of wild strain, △*qseC* and C-△*qseC*. Lane 1: the wild strain, Lane 2: the △*qseC*, Lane 3: C-△*qseC*. Lane M: protein molecular marker.
